# International expert opinion on optimal treatment of anastomotic leakage after rectal cancer resection: a case-vignette study

**DOI:** 10.1007/s00384-022-04240-5

**Published:** 2022-08-24

**Authors:** Kevin Talboom, Nynke G. Greijdanus, Frans van Workum, Sander Ubels, Camiel Rosman, Roel Hompes, Johannes H. W. de Wilt, Pieter J. Tanis, the TENTACLE-Rectum working group

**Affiliations:** 1 Department of Surgery, Amsterdam University Medical Centers, University of Amsterdam, Amsterdam, the Netherlands; 2 Department of Surgery, Radboud University Medical Centre, Radboud Institute for Health Sciences, Nijmegen, the Netherlands; 3 Department of Surgical Oncology and Gastrointestinal Surgery, Erasmus Medical Center, Rotterdam, the Netherlands

**Keywords:** Rectal cancer, Anastomotic leakage, Treatment

## Abstract

**Purpose:**

Little is known about the optimal treatment of anastomotic leakage after low anterior resection (LAR) for rectal cancer and whether treatment strategy depends on leakage features and patient characteristics. The objective of this study was to determine which treatment principles are used by expert colorectal surgeons worldwide.

**Methods:**

In this international case-vignette study, participants completed a survey on their preferred treatment for 11 clinical cases with varying leakage features and two patient scenarios depending on surgical risk (a total of 22 cases).

**Results:**

In total, 42 of 64 invited surgeons completed the survey from 18 countries worldwide. The majority worked at a university training hospital (62%) and had more than 15 years of experience performing LAR for rectal cancer (52%). Early leaks in septic patients were preferably treated by major salvage surgery, to some extent depending on the patient scenario. In early leaks in non-septic patients, drainage and faecal diversion were the cornerstones of the proposed treatment. Endoscopic vacuum therapy was more often proposed than percutaneous drainage. A minority proposed anastomotic reconstruction, more often for larger defects. Treatment of late leaks ranged from watchful waiting, drainage, or transanal repair to major (non-)restorative salvage surgery, with minimal influence of the degree of symptoms on the proposed strategy. Leaks of the blind loop and rectovaginal fistulae showed high variability in the proposed treatment strategy.

**Conclusion:**

This TENTACLE-Rectum case-vignette study demonstrates tailored treatment strategies depending on the clinical type of leak and patient characteristics, with variable degrees of consensus and knowledge gaps which should be addressed in future studies.

**Supplementary Information:**

The online version contains supplementary material available at 10.1007/s00384-022-04240-5.

## Introduction

Anastomotic leakage (AL) after LAR for rectal cancer remains a highly prevalent complication with serious consequences and leads to increased morbidity, increased risk of reinterventions, increased 90-day mortality in elderly patients, increased permanent stoma rates, and decreased quality of life. [1–6] In contrast to numerous studies on risk factors and prevention of anastomotic leakage, little is known about how to effectively treat AL after LAR.

Conventionally, AL after LAR is treated by dismantling the anastomosis or creating a diverting stoma (if not already present) and radiological or manual drainage of presacral collections [1]. More recently, new techniques have emerged such as endoscopic vacuum therapy (EVT), where a negative-pressure sponge is placed endoscopically into the presacral cavity. [7] EVT can be combined with a transanal closure of the defect (endoscopic vacuum-assisted surgical closure (EVASC)). [8] For certain types of leaks, such as a rectovaginal fistula or an ischaemic afferent colon, major reconstructive surgery can be considered: immediate redo anastomosis, delayed redo (Turnbull–Cutait), or intersphincteric proctectomy with complete debridement and pelvic cavity filling (e.g. omentoplasty).

The wide variety in applied treatment approaches is likely related to the clinical heterogeneity of AL after LAR. Different clinical entities can be defined depending on the time interval until diagnosis, concomitant abdominal sepsis, presence of ischaemia, degree of dehiscence, leakage-related symptoms such as sacral pain, and other leakage features such as the presence of a fistula (e.g. to the vagina).

Scarcely available studies on the treatment of AL after LAR focus on the efficacy of a single modality in unspecified leaks. In clinical practice, one should probably define the treatment goal and select a corresponding treatment principle first and then choose the most-suited modality to achieve this. Key principles in the treatment of AL can be identified in addition to general supportive interventions (e.g. feeding, antibiotics): abscess drainage, faecal diversion, temporary takedown of the anastomosis, reconstruction of the anastomosis, watchful waiting (WW), and definitive salvage surgery. Focusing on treatment principles instead of individual modalities may give more insight into the question of how AL should be approached based on relevant clinical parameters.

The aim of this case-vignette study was to gain more insight into how an international group of expert colorectal surgeons approach AL after LAR for rectal cancer in general and to investigate how these surgeons might tailor their approach to different subtypes of AL based on several leakage and patient characteristics.

## Methods

### Study design

This was an international case-vignette study in which a group of international experts were invited to participate by completing an online survey about the treatment of AL after LAR for rectal cancer. Invited experts were either part of the steering committee of the TENTACLE-Rectum study or the international TaTME Guidance collaborative. [9, 10] Invited experts are all experienced colorectal surgeons performing TME surgery and being actively involved in relevant scientific projects and/or colorectal societies. This survey consisted of a short general questionnaire and case discussions. The latter part included 11 clinical cases with different leakage features, and each case was presented for two different scenarios reflecting patients with low and high surgical risk, respectively (a total of 22 cases). The survey can be found in Tables 1 and 2, and a summary of the clinical cases is presented in Table 3. The survey was collected through the online platform Pluvo (www.pluvo.com), and all answers were analyzed and reported anonymously.

**Table 1:** Surgeon details

**Question**	**Total cohort (*n* = 42)**
***1. Country of origin***	
Europe	25 (60%)
North America	7 (17%)
South America	5 (12%)
Oceania	3 (7%)
Asia	2 (5%)
***2. Type of Hospital***	
Academic training hospital	26 (62%)
General teaching hospital	10 (24%)
Cancer center	5 (12%)
General (non-teaching) hospital	1 (2%)
***3. Years of experience***	
0–5 years	4 (10%)
6–10 years	8 (19%)
11–15 years	8 (19%)
> 15 years	22 (52%)
***4. Anual LAR caseload hospital***	
0–49	16 (38%)
50–99	18 (43%)
100 or more	8 (19%)
***5. Therapeutic modalities used for treatment of anastomotic leakage***	
Ultrasound guided percutaneous drainage	27 (64%)
CT-guided transgluteal drainage	39 (93%)
Laparoscopic absess drainage with placement abd. drain	36 (86%)
Transanal drainage under general anesthesia and placement of catheter for further drainage and irrigation of cavity	33 (79%)
Endoscopic washout of the abscess cavity	25 (60%)
EVT	31 (74%)
Endoscopic vacuum assisted closure system (EVT + surgical closure defect)	19 (45%)
Endoscopic clipping (i.e., OVESCO)	4 (9%)
Examination/transanal drainage under anesthesia	25 (60%)
Other	5 (12%)
***6. Available transanal approaches?***	
TAMIS	36 (86%)
TEM	20 (48%)
Open transanal approach with retractor	37 (88%)
TEO	10 (24%)

*LAR* low anterior resection, *CT* computed tomography, *EVT* endoscopic vaccuum therapy, *TAMIS* transanal minimally invasive surgery, *TEM* transanal endoscopic microsurgery, *TEO* transanal endoscopic operation

**Table 2:** Preferences

**Question**	**Total cohort (*n* = 42)**
***7. Do you always obtain fecal diversion (if not already present) in case of AL?***	
Yes, always	12 (29%)
Almost always, except small leak	24 (57%)
Almost always, except EVT patients	2 (5%)
Other	2 (5%)
***8. How are you preferably approaching a pelvic (presacral) abscess?***	
Manual transanal drainage on the ward	5 (12%)
Transgluteal percutaneous drainage	26 (62%)
Laparoscopic transabdominal drainage	14 (33%)
Endoscopic drainage without vacuum therapy	6 (14%)
EVT	21 (50%)
Transabdominal percutaneous drainage	11 (26%)
EUA + transanal tube drainage	21 (50%)
***9. Do you have any experience with anastomotic reconstruction?***	
No	6 (14%)
Yes, transanal closure	30 (71%)
Yes, redo	30 (71%)
***10. How would you approach a substantial amount of abdominal free fluids on CT in a patient with an ileostomy and non-ischemic leaking anastomosis?***	
Radiological	11 (26%)
Laparoscopy	24 (57%)
Laparotomy	2 (5%)
***11. What is your preferred approach for fecal or purulent peritonitis?***	
Laparoscopy	29 (69%)
Laparotomy	12 (29%)

*AL* anastomotic leakage, *EVT* endoscopic vaccuum therapy, *EUA* examination under anesthesia

**Table 3:** Summary clinical cases

**Case**	**Day**	**Diversion**	**Symptoms**	**Summary presentation AL**
**1**	3	Yes	Septic	Ischaemia afferent colon
**2**	3	Yes	Septic	Complete dehiscence anastomosis (3 cm retraction)
**3**	5	No	Mild	Presacral collection (2 × 2 cm) + 1/3 defect on endoscopy
**4**	5	Yes	None	Presacral collection and contrast extravasation, no defect on endoscopy
**5**	5	No	Mild	Presacral collection and contrast extravasation, defect blind loop (2 × 2 cm)
**6**	5	Yes	Mild	Presacral collection and contrast extravasation, large defect (> 50%) without ischaemia
**7**	50	Yes	None	Contrast extravasation and a small presacral collection
**8**	50	Yes	None	Contrast extravasation, but no presacral collection
**9**	50	Yes	Mild	Pus and air discharge through the vagina, no other clinical symptoms
**10**	250	No	Pain and LARS	Day 80 after closure of diverting ileostomy. Sacral pain and severe LARS. Presacral collection, 25% defect on posterior side
**11**	250	No	Mild	Mild pain, flatulence and mucus per anum. Presacral collection of air, 25% defect posterior side

### Questionnaire

The general questionnaire contained questions about the participants and their institutional setting (country, type of hospital, experience, annual caseload), therapeutic modalities used for AL, available techniques for transanal surgery, general treatment principles (faecal diversion, preferred approach to drain a pelvic abscess or to treat abdominal free fluid and fecal/purulent peritonitis) and experience with anastomotic reconstruction.

### Clinical cases

Eleven clinical cases were formulated by the TENTACLE-Rectum study team, with the aim to provide a broad range of leakage features that were expected to influence treatment strategy. These features included time interval to the diagnosis of AL (e.g. early leak on day 5, late diagnosed leaks on day 50 and 250), degree of dehiscence, location of the leak, retraction of the afferent loop, vascularization, size of presacral collections, presence of contrast extravasation on imaging, clinical symptoms (e.g. pain or low anterior resection syndrome (LARS)), hemodynamic instability (septic patient), and presence of a diverting ileostomy. All cases were presented for two clinical risk scenarios, which were a fit young patient and an elderly frail patient with comorbidities. Participants were able to select multiple answers for each clinical case to ensure that choosing a combination of modalities was possible.

### Treatment principles

For each clinical case, participants were asked to choose the most suitable treatment principle(s):

- Drainage: interventions aimed to drain presacral collections, e.g. intermittent transanal drainage (i.e. endoscopic wash-out), percutaneous radiological drainage, EVT.
- Reconstruction: procedures to transanally close the defect (open surgical approach (just Lonestar), transanal minimally invasive surgery (TAMIS) approach, endoscopic clipping (e.g. OVESCO)) or redo anastomosis after resection of the leaking anastomosis (i.e. immediate or delayed (Turnball–Cutait)).
- Faecal diversion: temporary diversion (defunctioning ileostomy or colostomy).
- Anastomotic takedown with the possibility of secondary reconstruction: end-colostomy without removing a rectal stump, leaving the original anastomosis in place.
- Watchful waiting: awaiting secondary healing.
- Definitive salvage surgery without the possibility of secondary reconstruction: intersphincteric resection of the rectal stump/anastomosis with debridement of the pelvic cavity and presacral filling (omentoplasty, flaps).

### Analysis

Descriptive statistics were used for this explorative study to gain insight into different treatment strategies for AL after LAR. Proportions of selected treatment modalities by participants were presented for each clinical case and clinical risk scenario. Analyses were carried out with IBM SPSS statistics, version 26.0 (IBM, Corp Armonk, NY, USA).

## Results

### Part 1: questionnaire

Out of 64 invited participants, 42 experts filled out the survey from 18 countries worldwide (66%). Most respondents originated from Europe (*n* = 25), of which were 4 from the UK, and 4 were from the Netherlands. The majority worked at an academic teaching hospital (62%) and had more than 15 years of experience performing LAR for rectal cancer (52%). In 62% of the participants, the annual number of LAR performed was more than 50 procedures (Table 1).

Amongst available treatment modalities for AL in the respondent’s hospital (Table 1), CT-guided transgluteal drainage was most frequently reported (93%). EVT was also commonly available (74%), as well as some type of transanal platform (TAMIS 86%, transanal endoscopic microsurgery (TEM) 48%, open transanal approach with retractor 88%).

The personal preferences of the participants regarding the treatment of AL are displayed in Table 2. Ninety-one percent of respondents diverted the leaking anastomosis always, or almost always with a few exceptions (small leak or EVT). The preferred approach(es) to drain a pelvic (presacral) abscess was/were transgluteal percutaneous drainage in 62%, EVT in 50%, laparoscopic transabdominal drainage in 33% and transabdominal percutaneous drainage in 26%.

Of the participants, 6 (14%) had no experience with anastomotic reconstruction, 30 (71%) had experience with transanal closure, and 30 (71%) had experience with redo procedures during which a new anastomosis is constructed. Abdominal free fluids were preferably approached by laparoscopy (57%), followed by percutaneous drainage (26%). The preferred approach for faecal or purulent peritonitis was most often laparoscopic (69%).

### Part 2: clinical cases

**Fig. 1:**
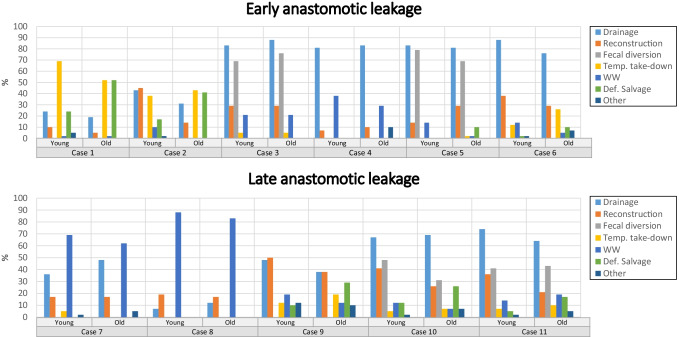
Proportions of proposed treatment modalities for each of the anastomotic leakage cases with two patient scenarios depending on surgical risk

Results from the clinical cases can be found for all leaks in Fig. 1, for early leaks in Table 4 and for late leaks in Table 5.

**Table 4:** Cases with early anastomotic leakage

	**Case 1**		**Case 2**		**Case 3**		**Case 4**		**Case 5**		**Case 6**	
	**Young**	**Old**	**Young**	**Old**	**Young**	**Old**	**Young**	**Old**	**Young**	**Old**	**Young**	**Old**
**Drainage**	10 (24%)	8 (19%)	18 (43%)	13 (31%)	35 (83%)	37 (88%)	34 (81%)	35 (83%)	35 (83%)	34 (81%)	37 (88%)	32 (76%)
Intermittent transanal drainage	6 (14%)	4 (10%)	7 (17%)	4 (10%)	11 (26%)	10 (24%)	5 (12%)	3 (7%)	11 (26%)	5 (12%)	10 (24%)	7 (17%)
Percutaneous radiological drainage	5 (12%)	5 (12%)	5 (12%)	8 (19%)	11 (26%)	11 (26%)	24 (57%)	24 (57%)	18 (43%)	17 (41%)	11 (26%)	13 (31%)
Endoscopic vacuum therapy (EVT)	0	2 (5%)	8 (19%)	6 (14%)	20 (48%)	23 (55%)	7 (17%)	7 (17%)	14 (33%)	17 (41%)	24 (57%)	19 (45%)
**Reconstruction**	4 (10%)	2 (5%)	19 (45%)	6 (14%)	12 (29%)	11 (26%)	3 (7%)	4 (10%)	6 (14%)	12 (29%)	16 (38%)	12 (29%)
Transanal surgical closure−open approach	0	0	5 (12%)	2 (5%)	7 (17%)	8 (19%)	3 (7%)	4 (10%)	1 (2%)	7 (17%)	11 (26%)	7 (17%)
Transanal surgical closure−TAMIS	1 (2%)	0	9 (21%)	3 (7%)	12 (29%)	10 (24%)	3 (7%)	4 (10%)	5 (12%)	11 (26%)	14 (33%)	9 (21%)
Transanal closure–endoscopic clipping	0	0	0	0	0	1 (2%)	0	0	0	2 (5%)	0	0
Immediate redo-anastomosis	4 (10%)	1 (2%)	6 (14%)	1 (2%)	1 (2%)	0	0	0	2 (5%)	2 (5%)	3 (7%)	2 (5%)
Delayed redo-anastomosis	3 (7%)	1 (2%)	8 (19%)	2 (5%)	0	0	0	0	0	0	1 (2%)	1 (2%)
**Fecal diversion**	NA	NA	NA	NA	32 (76%)	34 (81%)	NA	NA	33 (79%)	29 (69%)	NA	NA
Defunctioning ileostomy					29 (69%)	25 (60%)			30 (71%)	22 (52%)		
Defunctioning colostomy					3 (7%)	7 (17%)			2 (5%)	6 (14%)		
**Anastomotic take-down with possibility of secondary reconstruction**	29 (69%)	22 (52%)	16 (38%)	18 (43%)	2 (5%)	0	0	0	0	1 (2%)	5 (12%)	11 (26%)
**Watchful waiting**	1 (2%)	1 (2%)	4 (10%)	0	9 (21%)	2 (5%)	16 (38%)	12 (29%)	6 (14%)	1 (2%)	6 (14%)	2 (5%)
**Definitive salvage surgery without restoration of continuity**	10 (24%)	22 (52%)	7 (17%)	17 (41%)	0	0	0	0	0	4 (10%)	1 (2%)	4 (10%)
Intersphyncteric resection rectal stump/anastomosis	5 (12%)	16 (38%)	3 (7%)	13 (31%)	0	0	0	0	0	4 (10%)	0	4 (10%)
Debridement pelvic cavity	9 (21%)	17 (41%)	5 (12%)	13 (31%)	0	0	0	0	0	3 (7%)	1 (2%)	4 (10%)
Presacral filing with omentoplasty/flaps	1 (2%)	8 (19%)	1 (2%)	9 (21%)	0	0	0	0	0	3 (7%)	0	3 (7%)
**Other**	2 (5%)	0	1 (2%)	0	0	0	0	4 (10%)	0	0	1 (2%)	3 (7%)
**Combined modalities**												
Drainage and reconstruction	1 (2%)	0	10 (24%)	4 (10%)	12 (29%)	11 (26%)	3 (7%)	4 (10%)	4 (10%)	11 (26%)	15 (36%)	12 (29%)
EVASC (EVT with some form of transanal surgical closure)	0	0	3 (10%)	2 (5%)	8 (19%)	9 (21%)	1 (2%)	4 (10%)	2 (5%)	7 (17%)	11 (26%)	7 (17%)

*EVT* endoscopic vaccuum therapy, *TAMIS* transanal minimally invasive surgery

**Table 5:** Cases with late anastomotic leakage

	**Case 7**		**Case 8**		**Case 9**		**Case 10**		**Case 11**	
	**Young**	**Old**	**Young**	**Old**	**Young**	**Old**	**Young**	**Old**	**Young**	**Old**
**Drainage**	15 (36%)	20 (48%)	3 (7%)	5 (12%)	20 (48%)	16 (38%)	28 (67%)	29 (69%)	31 (74%)	27 (64%)
Intermittent transanal drainage	4 (10%)	6 (14%)	0	2 (5%)	8 (19%)	6 (14%)	6 (14%)	10 (24%)	8 (19%)	9 (21%)
Percutaneous radiological drainage	2 (5%)	5 (12%)	0	1 (2%)	5 (12%)	6 (14%)	10 (24%)	9 (21%)	6 (14%)	6 (14%)
Endoscopic vacuum therapy (EVT)	11 (26%)	11 (26%)	2 (5%)	4 (10%)	8 (19%)	7 (17%)	18 (43%)	16 (38%)	22 (52%)	22 (52%)
**Reconstruction**	7 (17%)	7 (17%)	8 (19%)	7 (17%)	21 (50%)	16 (38%)	17 (41%)	11 (26%)	15 (36%)	9 (21%)
Transanal surgical closure–open approach	5 (12%)	5 (12%)	5 (12%)	5 (12%)	8 (19%)	7 (17%)	6 (14%)	4 (10%)	8 (19%)	5 (12%)
Transanal surgical closure–TAMIS	5 (12%)	7 (17%)	8 (19%)	7 (17%)	8 (19%)	8 (19%)	7 (17%)	5 (12%)	8 (19%)	6 (14%)
Transanal closure–endoscopic clipping	0	0	1 (2%)	1 (2%)	1 (2%)	0	0	0	0	0
Immediate redo-anastomosis	2 (5%)	0	2 (5%)	0	7 (17%)	4 (10%)	10 (24%)	5 (12%)	6 (14%)	3 (7%)
Delayed redo-anastomosis	0	0	0	0	6 (14%)	4 (10%)	3 (7%)	3 (7%)	1 (2%)	2 (5%)
**Fecal diversion**	NA	NA	NA	NA	NA	NA	20 (48%)	13 (31%)	17 (41%)	18 (43%)
Defunctioning ileostomy							16 (38%)	10 (24%)	15 (35%)	13 (31%)
Defunctioning colostomy							2 (5%)	1 (2%)	1 (2%)	4 (10%)
**Anastomotic take-down with possibility of secondary reconstruction**	2 (5%)	0	0	0	5 (12%)	8 (19%)	2 (5%)	3 (7%)	3 (7%)	4 (10%)
**Watchful waiting**	29 (69%)	26 (62%)	37 (88%)	35 (83%)	8 (19%)	5 (12%)	5 (12%)	3 (7%)	6 (14%)	8 (19%)
**Definitive salvage surgery without restoration of continuity**	0	0	0	0	4 (10%)	12 (29%)	5 (12%)	11 (26%)	2 (5%)	7 (17%)
Intersphyncteric resection rectal stump/anastomosis					4 (10%)	12 (29%)	5 (12%)	10 (24%)	2 (5%)	7 (17%)
Debridement pelvic cavity					3 (7%)	7 (17%)	4 (10%)	10 (24%)	2 (5%)	7 (17%)
Presacral filing with omentoplasty/flaps					3 (7%)	6 (14%)	1 (2%)	9 (21%)	1 (2%)	6 (14%)
**Other**	1 (2%)	2 (5%)	0	0	5 (12%)	4 (10%)	1 (2%)	3 (7%)	1 (2%)	2 (5%)
**Combined modalities**										
Drainage + reconstruction	5 (12%)	7 (17%)	1 (2%)	4 (10%)	9 (21%)	8 (19%)	12 (29%)	9 (21%)	12 (29%)	8 (19%)
EVASC (EVT with TAMIS or open surgical closure)	3 (7%)	7 (17%)	1 (2%)	3 (7%)	3 (7%)	4 (10%)	4 (10%)	4 (10%)	8 (19%)	6 (14%)

*EVT* endoscopic vaccuum therapy, *TAMIS* transanal minimally invasive surgery

### Early leakage with sepsis (cases 1 and 2)

In a septic patient with an ischemic afferent colon (case 1), surgical modalities were preferred. Anastomotic takedown with the possibility of secondary reconstruction was chosen most often (69%). Definitive salvage surgery was chosen more often in the elderly frail patient compared to the young fit patient (52% vs 24%) and takedown with the possibility of secondary reconstruction was chosen less often in elderly frail patients (52% vs 69%).

In a septic patient with a completely dehiscent anastomosis (case 2), takedown with the possibility of secondary reconstruction was performed less often compared to case 1, with similar proportions for the two patient scenarios (38% in the young fit patient vs 43% in the elderly frail patient). Restorative treatment with anastomotic reconstruction was chosen in the young fit patient in 45%, and definitive salvage surgery was the main treatment approach in the elderly frail patient in 41%.

### Early leakage without sepsis (cases 3–6)

In a non-diverted patient with mild symptoms, a presacral collection and a defect (1/3 circumference) on endoscopy (case 3), the proposed treatment approach seemed to be independent of age and comorbidities, except for a higher proportion of watchful waiting in young fit patients (21% vs 5%). Most chose drainage (83% in the young fit patient and 88% in the elderly frail patient) combined with faecal diversion (76% and 81%, resp.). The preferred drainage modality was EVT (48% in the young fit patient and 55% in the elderly frail patient). The anastomosis would have been reconstructed by a minority of respondents (29% in the young fit patient and 26% in the elderly frail patient). In a diverted patient with an asymptomatic presacral collection without visible defect on endoscopy (case 4), most participants also chose drainage (81% in the young fit patient and 83% in the elderly frail patient), preferably by percutaneous drainage. EVT, as well as reconstruction, were less often proposed in case 4 as compared to case 3, with higher proportions of watchful waiting.

In the case of a non-diverted defect in the blind loop of a side-to-end anastomosis with mild symptoms (case 5), preferred treatment was comparable to case 3, although a diverting stoma was slightly less often proposed in the elderly frail patient. Reconstruction was less often preferred for defects of the blind loop in younger patients, compared to the elderly frail patient (14% vs 29%). Independent of patient scenario, less often EVT and more often percutaneous drainage was preferred for a blind loop defect as compared to a defect of the circular anastomosis.

If a mild symptomatic large defect is seen on endoscopy (> 50% of circumference) with a primary defunctioning stoma in situ (case 6), temporary takedown of the anastomosis was more often chosen compared to case 3, especially in the elderly frail patient (26% vs 0%). Also, more often, transanal surgical closure was considered than for case 3 (38% in the young fit patient and 29% in the elderly frail patient). The mainstay of treatment remained drainage (88% in the young fit patient and 76% in the elderly frail patient).

### Late leakage (cases 7–11)

Cases 7 and 8 are patients with a late radiological diagnosis of a diverted asymptomatic leakage based on contrast extravasation (postoperative day 50), with (case 7) or without (case 8) presacral collection. In contrast to early leaks, watchful waiting was most often proposed for both the young fit and elderly frail patient, especially in the absence of a presacral collection: 69% and 62% for case 7 and 88% and 83% for case 8, respectively. Drainage of a presacral collection in such occult leaks would not have been performed by the majority of respondents, with even lower proportions of reconstruction.

In a patient with a diverted rectovaginal fistula (case 9), surgical intervention would be performed more often when compared to the asymptomatic late leaks (case 7, 8). The preferred surgical strategy in the young fit patient was any type of reconstruction (50%) with a less often anastomotic takedown with a possibility of secondary reconstruction (12%) and definitive salvage surgery without restoration of continuity (10%). Corresponding proportions in the elderly frail patient were 38%, 19%, and 29%. Some would wait for the fistula to heal by itself. Many respondents asked for further information on the location and size of the defect.

In a patient with a secondary leak after stoma closure presenting with sacral pain and severe LARS (case 10), the treatment approach included drainage in two-thirds of respondents (67% in the young fit patients and 69% in the elderly frail patient) and less frequently faecal diversion (48% and 31%). Also, many surgeons would perform any surgical intervention to treat the leak itself, consisting of reconstruction (41% and 26%), anastomotic takedown with a possibility of secondary reconstruction (5% and 7%), and definitive salvage surgery without restoration of continuity (12% and 26%). A minority of participants chose watchful waiting in such a patient with sacral pain and severe LARS (12%). Case 11 represents an almost chronic leak with bowel continuity and mild symptoms. Proposed treatment strategies were comparable with those for case 10.

### Preferred type of treatment

Regarding drainage of presacral collections, EVT was the preferred strategy among the participants, with small differences depending on the indication. Percutaneous radiological drainage and intermittent transanal irrigation were second and third choices, with comparable proportions in most of the cases. If faecal diversion was chosen, predominantly a diverting ileostomy would be created instead of a colostomy (e.g. case 3 (69% vs 7%) or case 10 (38% vs 5%)). If transanal surgical closure was proposed, this would have been performed either by an open technique or by TAMIS in similar proportions.

## Discussion

This case-vignette study shows that proposed treatment strategies for AL after LAR for rectal cancer differed substantially depending on clinical presentation, leakage features and patient characteristics. A variable degree of consensus among the experts was observed. In addition to supportive care, drainage and faecal diversion are still considered to be the two main modalities of treatment, with a preference for active drainage using EVT among the participating surgeons. Among the minority who proposed surgical interventions, a wide variety in preferences for transanal repairs, dismantling of the anastomosis, and definitive salvage surgery was found. The results of this survey point towards several knowledge gaps.

The proposed treatment strategies with tailoring to the different clinical cases revealed some general principles as reflected by high consensus among the participating surgeons. Surgical treatment of the leakage was generally reserved for patients with severe acute leakage in accordance with a published Delphi consensus [11]. Transanal repair of the anastomosis or complete redo-anastomosis were infrequently used.

Besides these common practices, there were remarkable differences in surgeon preference in some cases. Some surgeons still relied on drainage in a septic patient with ischaemic or completely retracted afferent colon. One might question whether this results in the adequate control of sepsis, especially since passive drainage was the proposed modality (e.g. intermittent transanal or percutaneous drainage). Probably, pelvic drainage in such a patient can be used as a bridge to major salvage surgery, but active drainage with EVT might then be more effective on theoretical grounds. However, EVT is not available in every hospital, which might be the reason for using other drainage modalities. The optimal timing of salvage surgery and the role of bridging strategies with EVT to reduce the morbidity of major acute surgery are interesting fields of research to explore.

In patients with an early leakage, no sepsis and a small-sized defect, drainage, and faecal diversion (if applicable) was the preferred strategy of the respondents. Direct reconstruction was attempted in 7–39%, often combined with a drainage procedure. Interestingly, for similar patients with a larger defect (> 50%), a greater proportion of respondents would have attempted a direct closure method. Although a greater defect size might reduce the probability of leak healing without interventions aimed at repairing the defect, larger defects are also more difficult to close. In addition, traction on the anastomosis is more likely to be an explanatory component in larger leakages, which could hamper defect closure. A redo anastomosis, which has the potential to keep continuity without the drawbacks of defect closure may be an alternative and was indeed chosen by a limited number of respondents.

The leakage of the blind loop seems to be a distinct leakage entity after LAR, although seldom described in the literature. Blind loop leakage can be more difficult to drain effectively, and attempts of transanal or transabdominal closure are likely to fail based on personal experiences. These leaks appear to be prognostically worse with a lower chance of successful secondary healing. Hypothetically, intraluminal pressure within the blind loop can become high with peristaltic contractions in the presence of a competent internal sphincter, which probably explains the low chance of healing by any modality. This theory would argue in favour of major salvage surgery, but this is not confirmed by the present survey. Performing focus group discussions on leakage of the blind loop or collecting such cases in large multicentre collaborative research enabling pooled analyses, would likely provide more insight into this entity.

For an acute leak with a collection but no defect visible on endoscopy (case 4), most respondents chose percutaneous drainage and less frequently EVT or intermittent transanal drainage. The latter options require trans-anastomotic access. If there is an acute leak with a collection, one might be able to identify a small area of granulation tissue with an underlying small defect. Endoscopic probing of the anastomosis using a guide wire or biopsy forceps can help in identifying occult defects, which can subsequently be dilated. Expanding such a tiny defect often feels like aggravating the problem, which probably explains the clear preference of the participating surgeons for percutaneous drainage. Which strategy results in the highest chance of anastomotic integrity, in the end, is another interesting knowledge gap.

It is remarkable that drainage was still proposed by a substantial number of participants in late leaks. A pelvic abscess will generally induce extensive fibrosis around it. Collections diagnosed beyond the first few weeks are less likely to collapse by drainage as a result of this fibrosis formation with less pliability of surrounding tissues as a result. Even active drainage using EVT seems less successful in case of late initiation of treatment. [8] The GRECCAR group, which looked at EVT without transanal closure for AL, found a much higher restored continuity rate if treatment was initiated in the first 15 days after surgery (72% vs 28%). [12] The value of drainage procedures in late leaks as either a single modality or as a bridge to surgical interventions has still to be defined. Regarding the minority of participants proposing transanal closure of a late leak, the chance of success might be low when attempting to approximate the fibrotic edges of the two bowel ends together with stitches.

In a patient with a rectovaginal fistula (case 9), there was a large variety in chosen modalities and many participants indicated that they would like to know more details on the size and location of the defect. The preference for surgical interventions of the participants is likely explained by the presumed low chance of spontaneous healing because the fistula becomes the route of least resistance. Drainage is often difficult because generally, no collections build up. Whether specific details of the rectovaginal fistula should guide (type of) surgical intervention is unclear. In general, this less common presentation of AL is associated with many interventions, a significant impact on quality of life and a high rate of definitive salvage surgery, and available literature remains scarce [13, 14].

Symptoms of late leaks often consist of major LARS and sacral pain. Symptoms of frequent defecation will logically improve with faecal diversion, but chronic pelvic sepsis likely persists and can even worsen over time. Therefore, faecal diversion is not expected to reduce sacral pain. In case of severe symptoms, major salvage surgery might be the best option, but this was only chosen by a minority of participants. A reserved attitude towards major salvage surgery can be explained by the surgical complexity as well as the high risk of complications and need for reinterventions, with poor functional outcomes in case of redo anastomosis. [15, 16] Remarkably, an almost asymptomatic leak (case 11) was similarly treated as a very symptomatic leak (case 10).

The two different patient scenarios (the young fit or the elderly frail patient) did not appear to have much impact on decision-making in general, which is an interesting finding. Nevertheless, some exceptions were found. For example, participants were more likely to wait for secondary healing in young fit patients with early leaks, with slightly more definitive salvage surgery in elderly frail patients. In the absence of any evidence, one might also propose a more proactive surgical strategy for a young and fit patient to maximize the chances of preserving the anastomosis. Whether age and clinical condition should guide a treatment strategy also deserves attention in future studies.

This study has several limitations. Patient preference and shared decision-making are not included. Some patients may opt for a definitive stoma to prevent an extended treatment period, and if patients are unmotivated or unfit, this can alter the decision for a treatment option. We also did not focus on possible delays in treatment. Some participants commented that in some cases, they would first wait several months before attempting major reconstructive surgery. There is also a potential bias in how some treatment options were described with the unclarity of the used terms. For example, “delayed redo-anastomosis” was defined as a two-step redo (Turnbull–Cutait procedure) but might have been interpreted as a redo-anastomosis several weeks or months after diagnosis of the leak. We were not able to find clear differences in treatment approaches between countries or continents, but the participants might not have been representative of their countries. Finally, the exact location of the leak was not taken into account, and some treatment modalities might be more suitable for certain locations. For example, EVT is easier to apply for posterior leaks because there is more space compared to the anterior side. The location might be another variable to explore in future studies.

## Conclusion

This case-vignette study showed that proposed treatment modalities and principles for AL after rectal cancer are influenced by clinical leak presentation and patient characteristics. The heterogeneity of strategies to treat different cases of AL underlines the need for more clinical data on what strategies work for which patients with particular leakage characteristics.

## Supplementary Information

Below is the link to the electronic supplementary material.

Supplementary file1 (DOCX 13 KB)
