# Supplementary material for: International expert opinion on optimal treatment of anastomotic leakage after rectal cancer resection: a case-vignette study
Source: Int J Colorectal Dis. 2022 Aug 24;37(9):2049–59. doi: 10.1007/s00384-022-04240-5 (PMC9436864; doi:10.1007/s00384-022-04240-5)
Supplement: Supplementary file 1 — Supplementary file1 (DOCX 13 KB) [file 384_2022_4240_MOESM1_ESM.docx]

Supplementary table 1: country or origin participants

| **Country of origin** | **n** | **%** |
| --- | --- | --- |
| USA | 5 | 12% |
| Australia | 4 | 10% |
| Netherlands | 4 | 10% |
| UK | 4 | 10% |
| Italy | 3 | 7% |
| Spain | 3 | 7% |
| Switzerland | 3 | 7% |
| Austria | 2 | 5% |
| Belgium | 2 | 5% |
| Brasil | 2 | 5% |
| Canada | 2 | 5% |
| France | 2 | 5% |
| Argentina | 1 | 2% |
| Japan | 1 | 2% |
| New Zealand | 1 | 2% |
| NL | 1 | 2% |
| South Korea | 1 | 2% |
| Sweden | 1 | 2% |
